# Pharmacological principles for safe benzodiazepine and Z-drug dose reduction

**DOI:** 10.1017/S0033291726104887

**Published:** 2026-06-18

**Authors:** Mark Horowitz, Nicole Lamberson, Jaden Brandt, Adele Framer, Bryan Shapiro, Anders Sørensen, Cathal Cadogan, Alexis Ritvo, David Taylor

**Affiliations:** 1 North East London NHS Foundation Trust, Ilford, UK; 2 Benzodiazepine Information Coalition, USA; 3 College of Pharmacy, University of Manitoba, Winnipeg, MB, Canada; 4 Psychotropic Deprescribing Council, USA; 5 UC Irvine: University of California Irvine, USA; 6 Rigshospitalet Copenhagen Trial Unit, Denmark; 7 School of Pharmacy and Pharmaceutical Sciences, Trinity College Dublin, Ireland; 8 Department of Psychiatry, University of Colorado School of Medicine, USA; 9 King’s college, London, UK

**Keywords:** benzodiazepine-induced neurological dysfunction (BIND), benzodiazepine withdrawal, hyperbolic tapering, protracted withdrawal, receptor occupancy

## Abstract

Many guidelines recommend deprescribing benzodiazepines and z-drugs in most long-term users. Due to physical dependence in those using medication as prescribed (distinct from addiction), discontinuation can be difficult and is often unsuccessful, as withdrawal symptoms can be severe and long-lasting, especially after long-term use. There is a lack of clarity on how to taper patients in a tolerable manner that minimizes discomfort. Current guidelines vary with some suggesting linear reductions (e.g. 1 mg every 1–4 weeks) and some proportionate reductions (e.g. 5%–10% of the most recent dose per month, with smaller reductions as the dose decreases). The shape of the relationship between dose of benzodiazepine and activity at gamma-aminobutyric acid-A (GABA-A) receptors is hyperbolic: steeply inclining at low doses, flattening at higher doses. Consequently, linear dose reductions produce increasingly large changes in receptor occupancy, predicting escalating withdrawal effects, while hyperbolic or proportionate dose reductions produce linear reductions in pharmacological effect. Slower tapering (over months and years) may be more successful than tapering over days or weeks. In practice, rate of tapering should be adjusted according to severity of withdrawal effects. Final doses for some people will need to be very small (equivalent to as little as 0.2 mg of diazepam, or less) so that the last ‘step down’ to zero is not larger than prior tolerated reductions in terms of pharmacological effects. Liquids or compounded formulations of medication can be helpful at low doses to make small reductions. Guidelines should recommend hyperbolic tapering while awaiting randomized trials comparing linear with hyperbolic tapering.

## Introduction

While the benefits of benzodiazepines in acute syndromes are unchallenged for psychiatric indications (Krystal et al., 2003; Roth et al., 2005), the dangers of long-term prescribing are widely accepted (Olfson, King, & Schoenbaum, 2015). Consequently, most clinical guidelines recommend that benzodiazepines and z-drugs should be used only for acute situations, and not for longer than 2–4 weeks, to avoid the risk of dependence and withdrawal (Conn et al., 2020; Kaiser Permanente, 2019; National Institute for Health and Clinical Excellence, 2011). Longer-term use is associated with a number of physical and cognitive risks, including physical dependence, tolerance, loss of efficacy, falls, and fractures ( Donnelly et al., 2017) and memory disturbance (Lister, 1985). There is mixed evidence about the connection between benzodiazepine use and increased risk of dementia (De Gage et al., 2014; Friesen et al., 2025; Gray et al., 2016) and increased mortality (Ng, Le Couteur, & Hilmer, 2018; Patorno et al., 2017). Many clinical guidelines recommend that most long-term users should have benzodiazepines discontinued because of this unfavorable balance of benefits and harms (Brandt et al., 2024; Kaiser Permanente, 2019; Nice, 2018).

One in eight adults (12.5%) in the US takes benzodiazepines (Maust, Lin, & Blow, 2019), with about one-quarter of this use being long term (>4 months) (Olfson et al., 2015), equating to about 8 million Americans on long-term benzodiazepines. In England about 3% of the adult population took a benzodiazepine in 2017/2018(Public Health England, 2019), with about 0.7% taking benzodiazepines or z-drugs long term, equivalent to about 300,000 people (Davies, Rae, & Montagu, 2017).

One of the major barriers to stopping benzodiazepines is withdrawal effects, as emphasized by a ‘Boxed Warning’ FDA update to benzodiazepines about ‘the serious risks of abuse, addiction, physical dependence, and withdrawal reactions’ (FDA Drug Safety Communication, 2020). The FDA explained that previous prescribing information did not adequately warn about benzodiazepine’s serious risks and harms, meaning these medications might be prescribed ‘inappropriately’ (FDA Drug Safety Communication, 2020).

All guidelines recommend against stopping benzodiazepines abruptly or reducing the dosage too quickly because of withdrawal reactions, including seizures, which can be life-threatening (FDA Drug Safety Communication, 2020). Guidelines universally recommend tapering benzodiazepines, yet there is no definitive method, and most advice is based on expert consensus rather than empirical evidence or pharmacological principles, resulting in conflicting advice (Brunner et al., 2025; Pollmann, Murphy, Bergman, & Gardner, 2015).

Physicians are often hesitant to deprescribe in general, citing barriers such as lacking relevant skills and knowledge to deprescribe in a safe and effective manner, a lack of time and support, and fear of withdrawal or rebound reactions (Chouinard, 2004; Harriman, Howard, & McCracken, 2014). Patients also experience the withdrawal process as a difficult, complicated, and highly unpredictable process (Liebrenz, Gehring, Buadze, & Caflisch, 2015), some reporting that benzodiazepines are harder to discontinue than opioids because of the withdrawal symptoms (Liebrenz et al., 2015).

**Figure 1.**
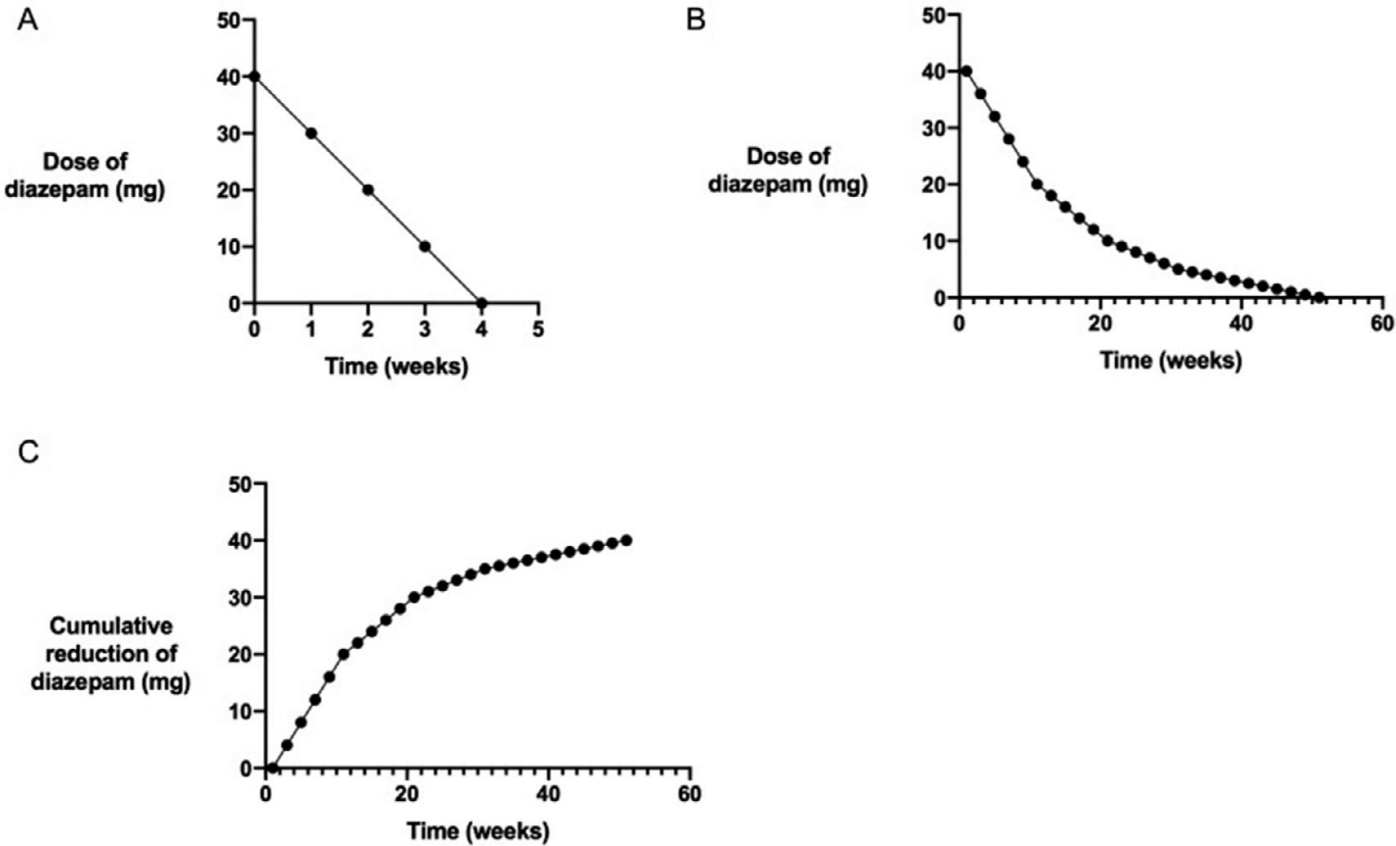
Different approaches to benzodiazepine tapering regimens recommended by NICE approximate hyperbolically reducing schedules. (a) A linear pattern of dose reduction is shown, involving a fixed percentage (in this case 25%) of the original dose reduced every week, as in many guidelines (McCormack et al., 2015; Permanente, 2019; Pottie et al., 2019). (b) The pattern of dose reduction recommended for tapering of diazepam from NICE and ASAM (for some patients) guidelines (of 5%–10% of the most recent dose each month) (Brunner et al., 2025; Nice, 2018). This pattern closely resembles a decreasing hyperbola. (c) cumulative reduction of diazepam per week as recommended by NICE guidance is represented (Nice, 2018). Note the similarity in the shape of this curve to the relationship between dose of benzodiazepines and both clinical and biological effects (other Figures). Figure 1. long description.

There are two main issues to consider with tapering – the rate at which tapering is performed (and so its overall duration) and the pattern of dose reductions. There are two principal patterns by which medications are tapered. A linear taper involves reduction of benzodiazepine dose by a fixed percentage of the original dose per time period – for example, 10%–25% of the original dose every 1–4 weeks (e.g. 1–2 mg of diazepam reduction every 1–4 weeks from a dose of 10 mg) (Kaiser Permanente, 2019; McCormack et al., 2015; Pottie et al., 2018) (Figure 1a). The second approach involves decreasing the dose by successively smaller amounts: for example, reducing dose by 5%–10% of *the most recent dose* (Box 1, Figures 1b–c), often referred to as proportionate tapering (Nice, 2018). This method of tapering approximates hyperbolic tapering where doses are reduced in such a way as to mirror the pattern of effect on target receptors (see further below).

Box 1. Suggested withdrawal schedule for diazepam (Nice, 2018), outlining a stepwise linear reduction regimen that approximates hyperbolic dose reduction

- Withdrawal should be gradual: such as 5–10% reductions every 1–2 weeks of the most recent dose, titrated according to the severity of withdrawal symptoms
- From diazepam 40 mg per day or less:
- Reduce dose by 2–4 mg every 1–2 weeks until reaching 20 mg per day, then
- Reduce dose by 1–2 mg every 1–2 weeks until reaching 10 mg per day, then
- Reduce dose by 1 mg every 1–2 weeks until reaching 5 mg per day, then
- Reduce dose by 0.5–1 mg every 1–2 weeks until completely stopped
- Estimated total withdrawal time:
- From diazepam 40 mg per day: 30–60 weeks
- From diazepam 20 mg per day: 20–40 weeks

**Table 1.** This table, based on the *E*_max_ equation of best fit derived from the data in Brouillet et al. (1991), after conversion to human equivalent dosage (Nair & Jacob, 2016), allows transformation from dosage of diazepam to indicator of biological effect, based on percentage occupancy of GABA-A Table 1. long description.

Diazepam dosage (mg)	GABA-A occupancy (%)^a^
200	89.0
100	80.2
75	75.2
50	66.9
37.5	60.3
25	50.3
12.5	33.6
10	28.8
5	16.8
2	7.5
1	3.9
0.5	2.0
0	0

a These doses are estimates based on averages, with limitations outlined in the text.

**Table 2.** This table demonstrates the doses required for reducing diazepam from 50 mg, in order to produce a linear reduction in biological effect (in this case approximately 1 percentage point decrements of estimated GABA-A occupancy) Table 2. long description.

Step	GABA-A occupancy (%)	Diazepam dose (mg)^a^	Step	GABA-A occupancy (%)	Diazepam dose (mg)^a^	Step	GABA-A occupancy (%)	Diazepam dose (mg)^a^	Step	GABA-A occupancy (%)	Diazepam dose (mg)^a^
1	66.9	50	22	49.3	24	43	31.8	11.5	64	12.1	3.4
2	66	48	23	48.2	23	44	30.8	11	65	11.5	3.2
3	65.1	46	24	47.1	22	45	29.8	10.5	66	10.8	3
4	64	44	25	46	21	46	28.8	10	67	10.2	2.8
5	63	42	26	44.7	20	47	28	9.6	68	9.5	2.6
6	61.8	40	27	44.1	19.5	48	27.1	9.2	69	8.9	2.4
7	61.2	39	28	43.5	19	49	26.3	8.8	70	8.2	2.2
8	60.6	38	29	42.8	18.5	50	25.4	8.4	71	7.5	2
9	60	37	30	42.2	18	51	24.5	8	72	6.8	1.8
10	59.3	36	31	41.5	17.5	52	23.5	7.6	73	6.1	1.6
11	58.6	35	32	40.8	17	53	22.6	7.2	74	5.4	1.4
12	57.9	34	33	40	16.5	54	21.6	6.8	75	4.6	1.2
13	57.2	33	34	39.3	16	55	20.6	6.4	76	3.9	1
14	56.4	32	35	38.6	15.5	56	19.5	6	77	3.1	0.8
15	55.7	31	36	37.8	15	57	18.5	5.6	78	2.4	0.6
16	54.8	30	37	37	14.5	58	17.4	5.2	79	1.6	0.4
17	54	29	38	36.2	14	59	16.3	4.8	80	0.8	0.2
18	53.1	28	39	35.3	13.5	60	15.1	4.4	81	0	0
19	52.2	27	40	34.5	13	61	13.9	4			
20	51.3	26	41	33.6	12.5	62	13.3	3.8			
21	50.3	**25**	42	32.7	12	63	12.7	3.6			

Some rounding of dose has been conducted to allow easier prescription using available tablets, and to restrict the need for liquid formulations until doses are less than 10 mg. These dosages could be prescribed with a combination of tablets and liquid formulations. Some patients may be able to tolerate this reduction schedule with steps spaced out every 2 to 4 weeks; others will require slower reduction schedules with intermediate steps inserted between the values given. The best guide to a tolerable rate of taper is the degree of withdrawal symptoms experienced by the patient. Some patients may benefit from twice-daily dosing of diazepam despite its long half-life probably because some people become sensitized even to small variation in plasma levels (Horowitz & Taylor, 2024). GABA-A occupancy is given to three significant figures. Further examples for hyperbolic tapering of all licensed benzodiazepines and z-drugs can be found in *The Maudsley Deprescribing Guidelines* (Horowitz & Taylor, 2024).

a These doses are estimates based on averages, with limitations outlined in the text.

**Table 3.** Tablet and liquid formulations available for commonly used benzodiazepines and z-drugs, with corresponding estimated GABA occupancy provided for one-quarter the lowest dose of available tablet Table 3. long description.

Benzodiazepine or z-drug^a^	Dose equivalent (Department of Health, 2017; Nice, 2018)	Elimination half-life (hours)[active metabolite](Ashton, 2002)	Tablet formulation available (Nice, 2018; RxList, 2020)	Liquid formulation available (s; RxList, 2020)	GABA occupancy of one-quarter the smallest tablet (%)
Alprazolam	0.25 mg	6–12	0.25 mg, 0.5 mg	1 mg/mL	4.8^b^
Chlordiazepoxide	12.5 mg	5–30 [36–200]	5 mg, 10 mg	N/A	2.0^b^
Clonazepam	0.25 mg	18–50	0.5 mg, 2 mg	0.5 mg/5 mL, 2 mg/5 mL	9.2^b^
Clorazepate	15 mg	20–160	US: 3.75 mg, 7.5 mg, 15 mg Europe:5 mg, 10 mg, 20 mg, 50 mg	N/A	US:2.5^b^ Europe:3.3^b^
Diazepam	5 mg	20–100 [36–200]	2 mg, 5 mg, 10 mg	2 mg/5 mL	2.0 (Brouillet et al., 1991; Nair & Jacob, 2016)
Eszopiclone	1 mg	6	1 mg, 2 mg, 3 mg	N/A	3.3^b^
Flurazepam	7.5–15 mg	[40–250]	15 mg, 30 mg	N/A	7.1^b^
Lorazepam	0.5 mg	10–20	0.5 g, 1 mg, 2.5 mg	1 mg/mL	4.8^b^
Lormetazepam	0.5 mg –1 mg	10–12	500 μg, 1 mg	N/A	4.8^b^
Nitrazepam	5 mg	15–38	5 mg	2.5 mg/5 mL	4.8^b^
Oxazepam	10 mg	4–15	10 mg, 15 mg	N/A	4.8^b^
Temazepam	10 mg	8–22	10 mg, 20 mg	10 mg/5 mL	4.8^b^
Triazolam	0.25 mg	1.5–5.5	0.125 mg 0.25 mg	N/A	3 (Bottlaender et al., 1994; Nair & Jacob, 2016)
Zaleplon	10 mg	1–1.5	5 mg, 10 mg	N/A	2.5^b^
Zolpidem	10 mg	2	5 mg, 10 mg	N/A	4.3 (Nair & Jacob, 2016; Schmid et al., 1995)
Zopiclone	7.5 mg	5–6	3.75 mg, 7.5 mg	N/A	1.4 (Benavides et al., 1992; Nair & Jacob, 2016)

a -Available in the UK or the US,

b - PET imaging is not available for these medications, and they have been calculated by converting to their diazepam equivalent (when equivalent dosing has a range, a range is reported)

## Methods

We conducted a narrative review of the benzodiazepine withdrawal syndrome and summarized commonly used tapering guidance from authorities in the UK and the US. We then reviewed the basic pharmacology of benzodiazepines, including the hyperbolic relationship between dose and their effects on the brain and behavior and the neurobiology underlying withdrawal. Targeted literature searches were conducted on PubMed and Google Scholar for the relevant domains. From this understanding we developed principles for tapering benzodiazepines based on fundamental pharmacology which may make the deprescribing process more tolerable for patients and improve rates of successful discontinuation.

This paper outlines principles for deprescribing benzodiazepines and z-drugs in patients who take them as prescribed – without benzodiazepine use disorder – which accounts for 98% of benzodiazepine users (Blanco et al., 2018). It distinguishes physical dependence – neuroadaptation causing withdrawal on reduction – from addiction, which also involves psychological dependence (including compulsion, craving, and other behavioral and cognitive components not seen in physical dependence alone) (Horowitz & Taylor, 2023; O’Brien, 2011). Severe withdrawal from physical dependence alone does not imply addiction. Management of addiction is outside this review’s scope.

## The benzodiazepine withdrawal syndrome

The FDA emphasizes that ‘physical dependence can occur when benzodiazepines are taken steadily for several days to weeks, even as prescribed’ (FDA Drug Safety Communication, 2020). Withdrawal symptoms from benzodiazepines can develop after just several days or weeks of continuous use and even after intermittent use (FDA Drug Safety Communication, 2020; Horowitz & Taylor, 2024). Normal-dose physical dependence was observed from the 1960s leading to their restriction by the FDA in 1975 (Guina & Merrill, 2018; Lader, 1991). In 1980 the Committee on the Review of Medicines in the UK focused on the issue of physical dependence and withdrawal and recommended that all benzodiazepine patients should have gradual withdrawal (Committee on the Review of Medicines, 1980). The 1980s and 1990s also saw regulatory restrictions for certain benzodiazepines (triazolam in particular) in Europe, Hong Kong, and the United States (Cloos et al., 2015). With the later entry of the z-drugs, manufacturers also placed warnings of tolerance, physical dependence, and withdrawal symptoms for these agents (Parsons, 2012).

The benzodiazepine withdrawal syndrome includes numerous physical and emotional symptoms due to the myriad effects of benzodiazepines on bodily systems (Box 2). As the withdrawal syndrome includes anxiety, panic, and insomnia, it may not only be misdiagnosed as a relapse of the original condition for which the benzodiazepine was prescribed but also a putative new-onset psychiatric disorder, thereby perpetuating unnecessary long-term treatment (Markota, Rummans, Bostwick, & Lapid, 2016; Moore, Pariente, & Begaud, 2015). Up to 90% of long-term users experience withdrawal effects on dosage reduction or stopping (Schweizer, Rickels, Case, & Greenblatt, 1990).

Box 2. The benzodiazepine withdrawal syndrome (Brunner et al., 2025; Cosci & Chouinard, 2020; Ng et al., 2018; Sokya, 2017)

**Figure:**
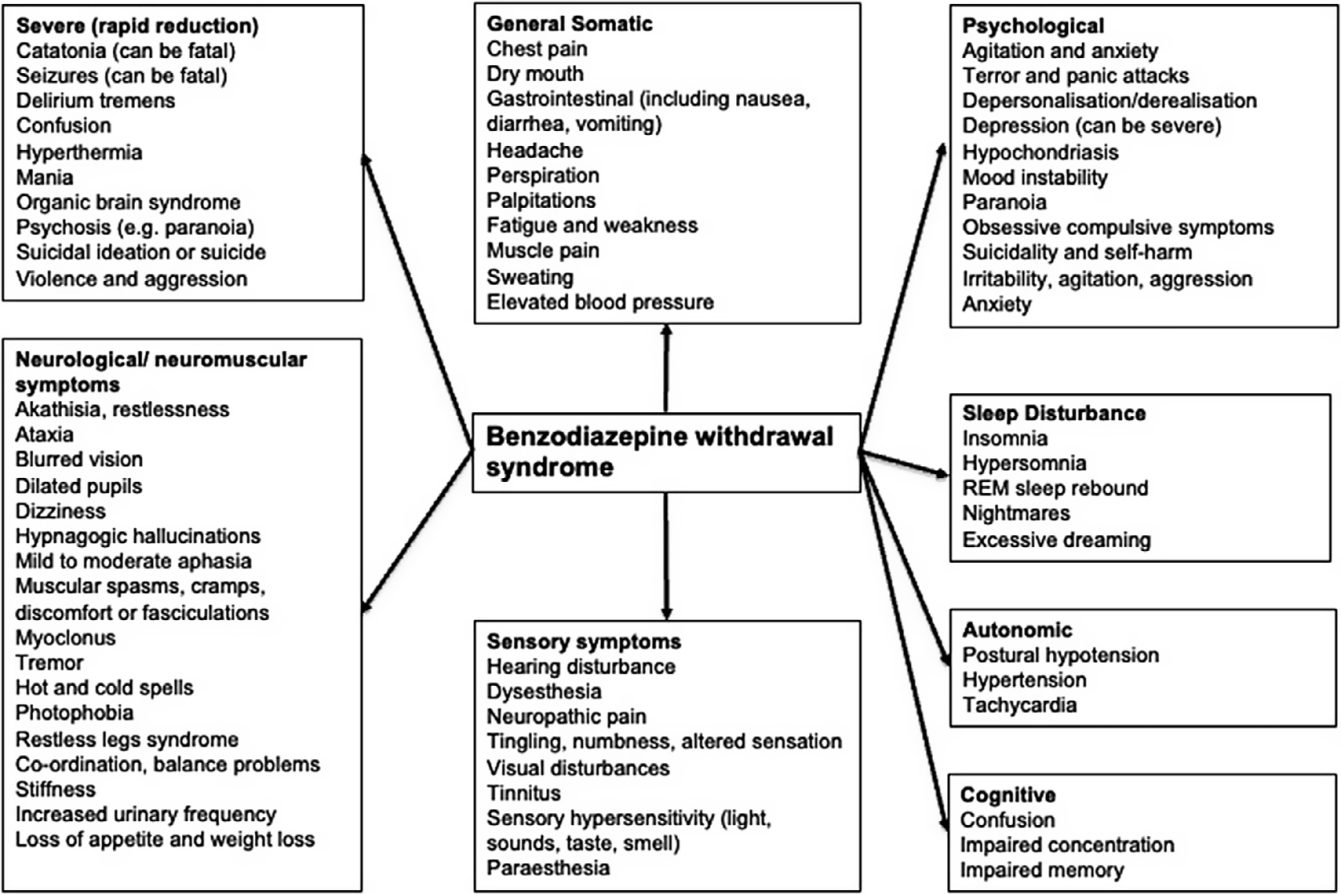
Box 2. long description.

Withdrawal effects have been classified in various ways, such as ‘new withdrawal symptoms’ (novel symptoms not experienced by the patient previously), ‘rebound symptoms’ (symptoms that the patient has experienced previously but with greater intensity), and ‘persistent post-withdrawal disorder’ (where withdrawal symptoms last for months or years after cessation) (Cosci & Chouinard, 2020). The FDA has emphasized that withdrawal effects can last from weeks to more than a year (Ashton, 1987; FDA Drug Safety Communication, 2020). Due to the persisting cognitive, affective, and somatic symptoms in these protracted withdrawal syndromes, experts have suggested the term ‘Benzodiazepine Induced Neurological Dysfunction’(BIND) as more appropriate, recognizing that such protracted symptoms often do not respond to re-instatement (Ritvo et al., 2023; Shade et al., 2025), like similar post-cessation-induced neurological dysfunction seen from other classes of psychiatric drugs (Horowitz & Davies, 2024). These protracted withdrawal syndromes have also been named ‘persistent post-withdrawal disorders’ by Chouinard and colleagues (Cosci & Chouinard, 2020).

## Pharmacokinetic and pharmacodynamic variation and effect on withdrawal

Withdrawal arises from both pharmacokinetic and pharmacodynamic adaptations and so withdrawal risk varies between benzodiazepines according to half-life, potency, lipophilicity, receptor affinity, and the presence of active metabolites (Shapiro, 2025; Teboul & Chouinard, 1990, 1991). Benzodiazepines with shorter elimination half-lives more commonly cause severe withdrawal problems (Chouinard, 2004; Horowitz & Taylor, 2024). Aspects such as the alpha half-life (the rate of decline in plasma concentrations due to the process of drug redistribution from the central to the peripheral compartment), derived from single dosing studies, may be even more influential on withdrawal risk. This is particularly the case for alprazolam and lorazepam, with short alpha half-lives (Teboul & Chouinard, 1990, 1991). High-potency benzodiazepines (greater receptor occupancy per milligram and tighter binding), such as alprazolam, clonazepam, and lorazepam, cause more withdrawal than lower-potency benzodiazepines (Chouinard, 2004). However, clonazepam’s favorable pharmacokinetic profile (longer alpha and beta half-lives) lends itself to less plasma fluctuations and inter-dose withdrawal anxiety despite its high potency (Teboul & Chouinard, 1991). Further risk factors for the withdrawal syndrome are shown in Box 3, including higher doses, longer duration of treatment, whether benzodiazepines have active metabolites with long half-lives, the presence of inter-dose withdrawal (Teboul & Chouinard, 1991), and a history of multiple drug switches or withdrawal attempts.

Box 3. Risk factors for benzodiazepine withdrawal (All Wales Medicines Strategy Group, 2016; Ford et al., n.d.; Horowitz & Taylor, 2024; Teboul & Chouinard, 1990, 1991)

- Drug dose, frequency, and duration of use – frequent, long-term, high-dose users at increased risk
- History of exposure to numerous psychiatric medications and multiple switches or cessation of these drugs (‘kindling’ phenomenon)
- Experience of inter-dose withdrawal while on stable dose
- Past history of withdrawal effects on missed doses or attempts at reduction/cessation
- Previous unsuccessful withdrawal attempts
- Use of benzodiazepines with high potency and short alpha (time to distribute drug from central compartment to peripheries) and beta (elimination) half-lives (such as alprazolam and lorazepam)
- Higher potency benzodiazepines greater receptor occupancy per mg of drug cause more withdrawal (e.g. alprazolam, clonazepam, and lorazepam)
- Benzodiazepines with long-acting active metabolites (e.g. diazepam) have a lower risk of withdrawal than those without
- A history of current or past alcohol or other sedative-hypnotic use
- Use of recreational drugs

## Linear tapering in clinical practice guidelines

In two scoping reviews of benzodiazepine tapering studies (Brandt et al., 2024; Pollmann et al., 2015), most original investigations and guidelines employed reductions of 10%–25% every 1–2 weeks in linear fashion (i.e. 75% of the original daily dose, then 50% of the original dose, 25% of the original dose), and then discontinuation (Pollmann et al., 2015). The final dose before complete cessation was generally 25% or 50% of the original dose, when reported, in these studies (Pollmann et al., 2015), with some implying a final dose of 5%–10% of the original dose (Brandt et al., 2024). Similarly, an influential clinical practice guideline recommends reducing the dose by ‘25% per week’ for rapid reduction and ‘10% every 2–4 weeks’ as a slower method (Kaiser Permanente, 2019). Notably, most guidelines recommend that percentage reductions all refer to the original dose of medication taken by the patient and therefore represent linear tapers (Pottie et al., 2019).

## Proportionate/hyperbolic tapering in clinical practice guidelines

In contrast, other guidelines (Ashton, 2002; Horowitz & Taylor, 2024; Nice, 2018) recommend tapering according to a proportionate pattern. For example, the NICE Clinical Knowledge Summary on benzodiazepines and z-drug withdrawal suggests that benzodiazepines are tapered at approximately 5%–10% of *the most recent daily dose* every 1–2 weeks, meaning that the size of the reductions becomes smaller and smaller as the total dose gets lower (Nice, 2018). When patients are taking 40 mg, ‘5%–10%’ corresponds to 2–4 mg every 1–2 weeks, but when down to 10 mg, this corresponds approximately to 1 mg every 1–2 weeks (Box 1) (Nice, 2018). Generally, 0.5–1 mg of diazepam is the final dose suggested by such tapering regimens before complete cessation (approximately 1%–2% of the original dose) (Ashton, 2005; Horowitz & Taylor, 2024). Similarly, the NICE guidelines on safe withdrawal of prescribed drugs of dependence recommend ‘a slow, stepwise rate of reduction proportionate to the existing dose, so that decrements become smaller as the dose is lowered’ (NICE, 2022).

In 2025, a joint clinical practice guideline on benzodiazepine tapering developed by the American Society of Addiction Medicine (ASAM), funded by the FDA, partnered with nine other medical societies including the American Psychiatric Association, recommended linear dose reductions as the preferred strategy (5%–10% of the daily dose every 2–4 weeks) (Brunner et al., 2025). However, the ASAM guideline also cites the *Maudsley Deprescribing Guidelines: Antidepressants, Benzodiazepines, Gabapentinoids and Z-drugs* to recommend hyperbolic tapering for patients who experience withdrawal symptoms in order to mitigate these effects (Brunner et al., 2025; Horowitz & Taylor, 2024).

## Existing evidence for linear versus hyperbolic tapering

There has been limited research evaluating these different methods of tapering. However, clinical studies have reported that linear tapering is unsuccessful for a significant proportion of patients. For instance, one trial found 90% of those who linearly tapered (25% of reduction per week from the original dose) experienced withdrawal symptoms, with 32% of those on long half-life benzodiazepines and 42% of those on short half-life benzodiazepines unable to cease their medication because of withdrawal symptoms (Schweizer et al., 1990). Only 36% of patients were able to successfully stop their benzodiazepine in a similar trial with a 4-week taper at 15-month follow-up (Voshaar et al., 2003).

A survey of specialized deprescribing clinics around the world report using hyperbolic tapering as their main approach to safely stopping psychiatric drugs, including benzodiazepines (Cooper et al., 2023). Consistent with this, a recent systematic review of tapering strategies for psychiatric drugs, including benzodiazepines, concluded that ‘hyperbolic tapering…appears to be the most promising strategy for psychiatric drug discontinuation’ (Eserian et al., 2023). Hyperbolic tapering has been adopted by the NICE guidelines for several other classes of drugs including opioids and antidepressants, and suggested for higher risk patients in antidepressant tapering guidelines in Australia and Canada, based on the biological plausibility of this approach and emerging empirical evidence(Horowitz & Wilcock, 2022; Horowitz et al., 2021; Horowitz & Taylor, 2021; Jauca, n.d.; NICE, 2022; Therapeutic Guidelines, 2025). However, currently there are no randomized controlled trials comparing linear tapering with hyperbolic tapering.

## Pharmacodynamics of benzodiazepines and z-drugs

Benzodiazepines and z-drugs are positive allosteric modulators of the GABA-A receptor, producing their clinical effects by enhancing the effect of the neurotransmitter GABA at the GABA-A receptor (Cheng, Wallace, Ponteri, & Tuli, 2018; Gunja, 2013). GABA is a universal inhibitor of neural activity and decreases the release of many neurotransmitters with excitatory properties (acetylcholine, noradrenaline, dopamine, serotonin, and glutamate), thought to produce the subjective experience of anxiolysis and sedation (Cheng et al., 2018; Turton & Lingford-Hughes, 2016).

The relationship between dose of benzodiazepines (and z-drugs) and their effect on GABA-A receptors is hyperbolic (Holford, 2018). This relationship arises as a consequence of the law of mass action, whereby additional drug molecules produce increasingly small additional effects as receptors become increasingly saturated (Holford, 2018). The hyperbolic nature of this relationship is often obscured by the common practice of plotting dose–response curves with dose on a logarithmic axis, yielding sigmoid curves with plots that appear linear at intermediate doses (Holford, 2018).

**Figure 2.**
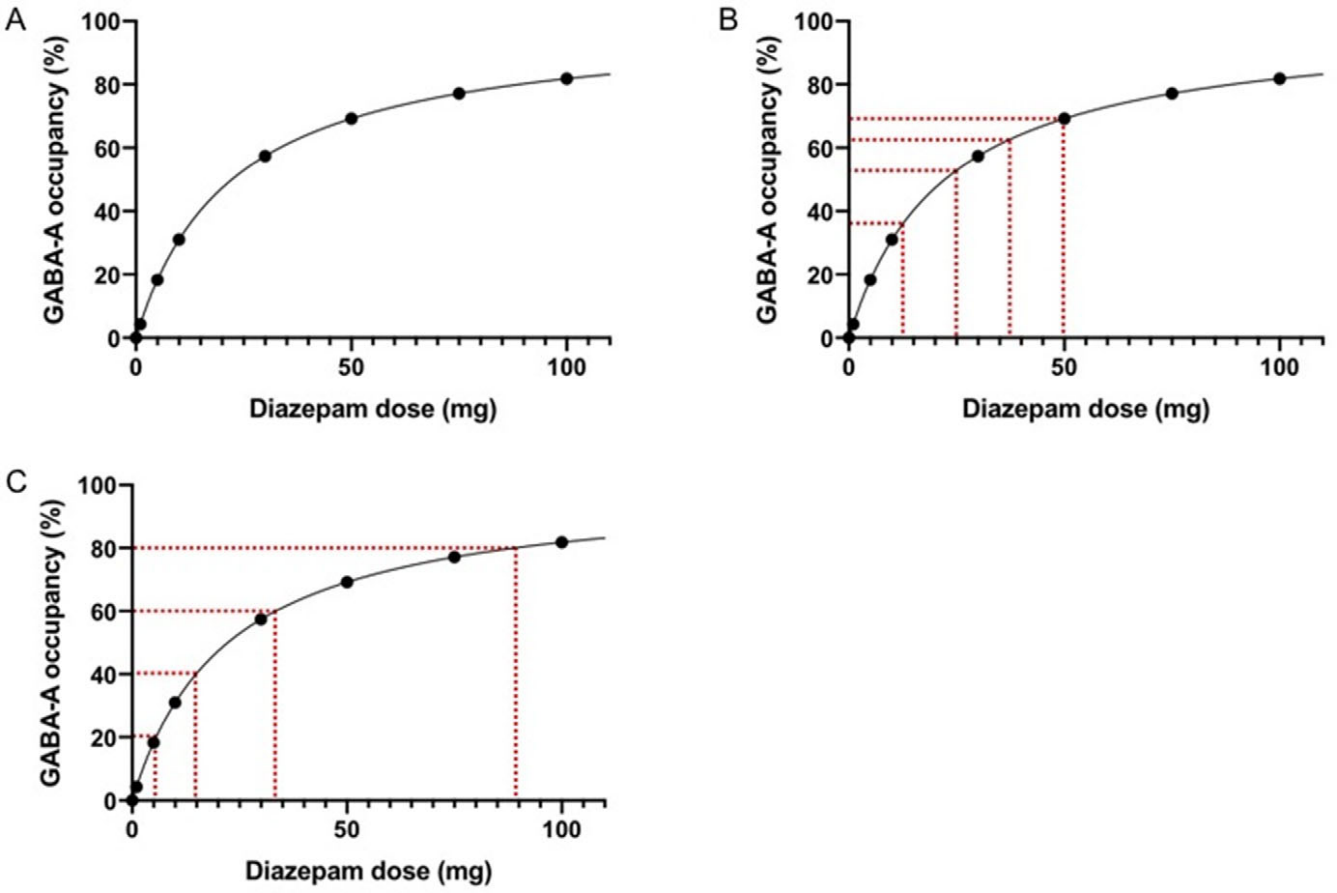
The differential effects of tapering benzodiazepines according to a linear or hyperbolic pattern of reduction. (a) The relationship between dose of diazepam and action at GABA-A receptors. Note the hyperbolic relationship between dose and estimated GABA-A occupancy. This graph was adapted from Brouillet et al. (1991), a study conducted in baboons, with dosages converted to human equivalents, as outlined in Nair and Jacob (2016). (b) Linear dose reductions of diazepam cause hyperbolically increasing reductions in effect at the GABA-A receptor. This may be associated with increasingly severe withdrawal effects. (c) Hyperbolically decreasing dose reductions produce linear reductions in effect at GABA-A receptors. This may be associated with more ‘evenly spread’ withdrawal effects. Figure 2. long description.

PET scanning, using the radioligand [^11^C]flumazenil in non-human primates (Figure 2a), allows visualization of this hyperbolic relationship (Atkins & Nimmo, 1975; Brouillet et al., 1991; Holford, 2018). This relationship is notable for the relatively shallow slope of the relationship between dose and GABA-A occupancy at high doses of diazepam, and the steep gradient at lower doses.

## Hyperbolic pattern in benzodiazepine effects

**Figure 3.**
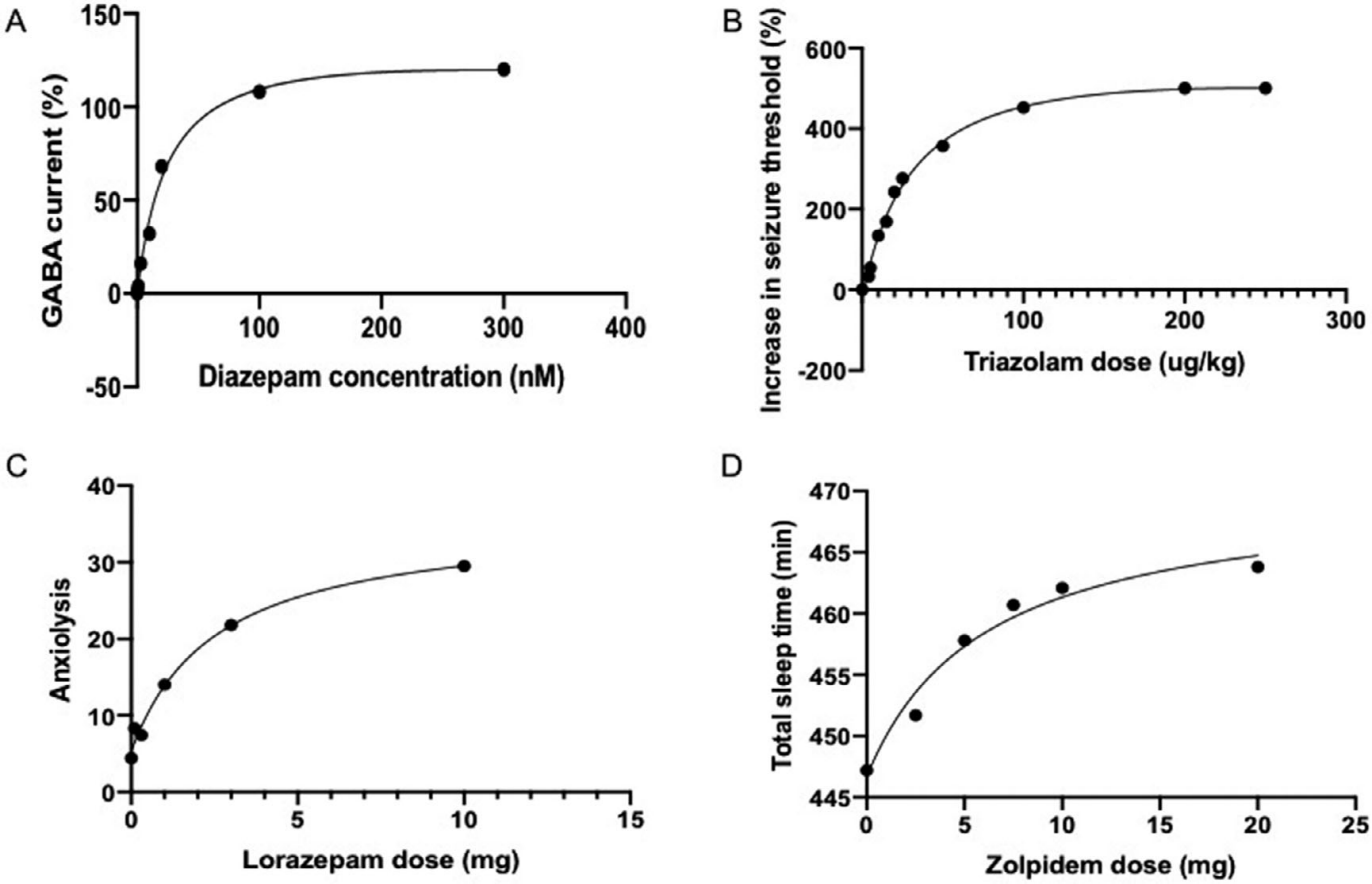
The relationship between dose of benzodiazepine or z-drug and many biological and clinical effects is hyperbolic. (a) Diazepam produces a hyperbolic relationship between benzodiazepine-site concentration and GABA current in human recombinant GABA-A receptors. Adapted from Atack (2009). (b) Triazolam produces a hyperbolic relationship between dose and increase in seizure threshold in non-human primates. Adapted from Bottlaender et al. (1994). (c) Lorazepam demonstrates a hyperbolic relationship between dose and degree of anxiolysis. Anxiolysis was measured by mean percentage of open arm entries on the elevated plus-maze in mice. Adapted from Dubinsky et al. (2002). (d) Zolpidem produces a hyperbolic relationship between dose and total sleep time in human subjects. Adapted from Merlotti et al. (1989). Figure 3. long description.

The hyperbolic dose–receptor occupancy relationship is mirrored in multiple neurochemical and behavioral effects, indicating clinical relevance. GABA-gated currents increase hyperbolically with increasing diazepam concentrations in cellular models (Berezhnoy et al., 2008; Wongsamitkul et al., 2017), including recombinant human GABA-A receptors (Atack, 2009) (Figure 3a). Seizure threshold shows a hyperbolic relationship to dose in zebrafish (Gupta, Khobragade, & Shingatgeri, 2014), rats (Kapur & Macdonald, 1997), and non-human primates (Bottlaender et al., 1994) (Figure 3b). Anxiolytic effects increase hyperbolically in pigeons (Kleven & Koek, 1999), rats (Dubinsky et al., 2002), and non-human primates (Rowlett et al., 2005) (Figure 3c). Z-drugs display hyperbolic effects on locomotion, seizure threshold, muscle relaxation, and ataxia in mice (Sanger & Depoortere, 1998).

In humans, hyperbolic relationships occur between benzodiazepine dose and saccadic slowing/sedation (Hommer et al., 1986), zolpidem’s effects on sleep (Merlotti et al., 1989) (Figure 3d), diazepam’s impact on cortisol and growth hormone (Hommer et al., 1986) and delirium risk in critically ill children (Mody et al., 2018).

Withdrawal symptoms also appear to be hyperbolically dose-dependent: larger reductions cause greater severity, particularly at lower doses (Ashton, 2004). For example, it has been observed that 1 mg reductions are generally tolerable from 20 mg diazepam but not from 5 mg, where smaller decrements are preferred (Ashton, 2004), suggesting a hyperbolic relationship between dose reduction and withdrawal severity.

## Neurobiology underpinning tapering

Adaptations to a substance cause tolerance during exposure and predict withdrawal when the substance is removed and these adaptations act unopposed (Turton & Lingford-Hughes, 2016). The physiological mechanisms of benzodiazepine tolerance and adaptation remain uncertain, with mixed findings on reduced number or sensitivity of GABA-A receptors (Cheng et al., 2018). Preclinical models suggest uncoupling – loss of allosteric modulation at the benzodiazepine site – as a likely mechanism (Cheng et al., 2018). Chronic benzodiazepine use also down-regulates adenosine receptors and up-regulates glutamatergic NMDA and AMPA receptors, likely compensating for enhanced GABA activity (Cheng et al., 2018).

Relative underactivity of inhibitory GABA activity, and its downstream effects, during benzodiazepine dose reduction likely cause withdrawal symptoms (Authier et al., 2009), supported by findings that administration of flumazenil – a short-acting selective GABA-A antagonist – induces rapid-onset withdrawal (Mintzer, Stoller, & Griffiths, 1999). Reduced GABA transmission allows increased excitatory activity, as neurotransmitters like glutamate act with less opposition (Ashton, 2005), perhaps explaining symptoms such as panic, anxiety, tachycardia, diaphoresis, and typical of excitatory states. Withdrawal likely resolves once receptor changes and downstream processes caused by adaptation to drug exposure sufficiently reverse (Ashton, 2005). Although not well understood, the rate of this reversal varies between individuals and with exposure duration, perhaps explaining withdrawal durations from weeks to years (Ashton, 1995; Lader & Morton, 1992).

The rationale for tapering is that gradually reducing GABA-A receptor modulation by benzodiazepines minimizes disturbance to homeostatic balance achieved at steady-state dosing and thus withdrawal symptoms. Slow tapering allows neuroadaptations to resolve in step with reduced receptor activation (Ashton, 2005; Horowitz & Taylor, 2021).

## Pharmacological rationale for hyperbolic tapering

Benzodiazepine dose demonstrates a hyperbolic relationship with both GABA-A occupancy and clinical effects. This suggests a linear relationship between GABA-A occupancy and clinical effects, supported by seizure-threshold studies in primates (Ashton, 2002; Framer, 2021; Nice, 2018). A similar linear relationship between withdrawal effects and GABA-A occupancy is plausible. To minimize withdrawal, especially at lower doses, GABA-A activity should be reduced linearly, which requires hyperbolically decreasing dose reductions. The benzodiazepine dose–response curve illustrates the impact of linear tapering (Figure 2a): reducing diazepam by fixed 12.5 mg intervals from 50 mg yields progressively larger drops in GABA-A occupancy – 6.6 percentage points (p.p.) (50 to 37.5 mg), 10.0 p.p. (37.5 to 25 mg), 16.7 p.p. (25 to 12.5 mg), and 33.6 p.p. (12.5 to 0 mg) (Figure 2b, Table 1). At low doses, small reductions have disproportionately large effects; for example, 5 to 0 mg changes occupancy by 16.8 p.p., exceeding the change from 100 to 50 mg (13.3 p.p.), consistent with the clinical observation of increasing withdrawal severity near zero (Ashton, 2004).

A regimen that reduces GABA-A occupancy linearly – e.g. 50 mg by 4 equal-sized steps of receptor occupancy – would entail 50 mg (66.9% occupancy), 24.9 mg (50.2%), 12.4 mg (33.5%), 5.0 mg (16.7%), and 0 mg (Figure 2c). A more gradual regimen reducing occupancy by approximately 1 percentage point at a time is shown in Table 2.

A hyperbolic reduction regimen can be closely approximated by an exponential one, which can be easier to calculate. For example, reducing the dose by 10% of the most recent dose at each step equates to approximately 2 percentage-point reductions in GABA-A occupancy, while 5% reductions equate to approximately 1 percentage point reductions. This seems to support the validity of the roughly exponential regimens (5%–10% reductions of the most recent dose) derived from clinical experience (Ashton, 2002; Framer, 2021; Nice, 2018).

This analysis suggests that final benzodiazepine doses before cessation should be far below therapeutic levels, so the last reduction to zero is no larger (in receptor occupancy terms) than previously tolerated steps. This aligns with regimens recommending final diazepam doses of 0.2–0.5 mg (1%–2% GABA-A occupancy), with some patients requiring even smaller doses (Ashton, 2005; Gupta, Cahill, & Miller, 2018).

## Limitations of the review

Several limitations should be noted when interpreting the evidence base for hyperbolic tapering of benzodiazepines.

The PET imaging data used to model the dose-occupancy curve come from non-human primates rather than humans; while receptor pharmacology is broadly comparable, species differences in subunit composition, distribution, and pharmacokinetics may exist (Atack et al., 2010; Friedman, Redmond, & Greenblatt, 1991). These data also represent single doses and population averages, which obscure inter-individual variability in receptor sensitivity, binding affinity, and pharmacodynamic response. No empirical studies have directly tested linear versus hyperbolic tapering; this remains a biologically plausible inference from receptor theory, supported by clinical experience, including patient-led experience. Modeling assumes steady-state pharmacokinetics and linear elimination, which may be disrupted by illness, polypharmacy, or metabolic variability.

Nonetheless, because pharmacodynamic relationships follow the law of mass action, the general shape of the dose-occupancy curve – dictated by ligand–receptor binding kinetics – is unlikely to differ substantially between primates and humans or across individuals, even if exact inflection points vary. Thus, while absolute parameters may shift, the hyperbolic nature of the relationship is unlikely to be an artifact, providing a sound rationale for hyperbolic tapering. In practice, individual differences are best managed empirically, using the intensity of withdrawal symptoms as the main guide to taper speed rather than a ‘one-size-fits-all’ approach.

## Implementation in clinical practice

### Rate of tapering

The effectiveness of different tapering rates is not well studied. Rapid withdrawal produces more severe symptoms and higher dropout rates than slower tapering (Morin, Bélanger, Bastien, & Vallières, 2005; Parr et al., 2008), while gradual reduction yields better outcomes (Parr et al., 2008). A systematic review of 35 studies found the best results with a 10-week taper, though 31% still could not stop (Cantopher, Olivieri, Cleave, & Edwards, 1990; Denis, Fatseas, Lavie, & Auriacombe, 2005). In another study, only 38% of patients on benzodiazepines for >3 months successfully discontinued after a 4–8-week taper (Baillargeon et al., 2003).

Although the half-lives of these drugs (most less than 24 hours, with some exceptions including commonly prescribed drugs like diazepam and clonazepam) may suggest that dose reductions could be made every few days, dose reduction intervals are better guided by the time needed for neuroadaptations to resolve rather than drug elimination (Reidenberg, 2011). Withdrawal symptoms can last for many months and sometimes years (Ashton, 1987, 2005; Barker, Greenwood, Jackson, & Crowe, 2004; Higgitt, Fonagy, Toone, & Shine, 1990), and meta-analysis shows cognitive effects after long-term use lasting over six months (Barker et al., 2004).

Therefore, tapering periods, especially for long-term users, might require months, and even years, to allow underlying neuroadaptations to resolve. A trial reduction of approximately 2% GABA-A occupancy (about 10% of the current dose) may be followed by 2–4 weeks of monitoring, resuming reductions only when symptom-free for at least a week. The *Maudsley Deprescribing Guidelines* (M. Horowitz & Taylor, 2024) provide drug-specific hyperbolic tapering schedules for all licensed benzodiazepines and z-drugs. In a large survey, self-paced tapers adjusted to withdrawal symptoms were linked to greater success (Lynch et al., 2024). If symptoms become unpleasant or severe, the taper may be paused or reversed, and then resumed at a slower rate (Horowitz & Taylor, 2024). Some may choose faster tapers despite more severe symptoms, though risks will be higher.

Various factors (Box 3) are thought to increase the risk of withdrawal effects such as benzodiazepines with shorter elimination and alpha (redistribution) half-lives, drugs with greater potency, those used in higher doses and for longer periods of time, and patients with evidence of inter-dose withdrawal and with past experiences of withdrawal effects on stopping or reducing their dose. The greater the risk of withdrawal, the more likely that gradual and hyperbolic dose reductions will be beneficial. The risk may be particularly high for triazolobenzodiazepines (like alprazolam and triazolam) which have the greatest withdrawal risk due to their lipophilicity, high potency, and high binding affinity at GABA-A receptors, and are the drugs most likely to be used in high doses for long periods (Cloos et al., 2015). Notably, the increased risk of withdrawal from short half-life benzodiazepines can be mitigated by multiple daily dosing so that daily plasma variations are minimized. Further research could help more precisely match taper rates to factors such as age, drug exposure duration, withdrawal history, co-morbidities, and elimination half-life.

Theoretically, z-drugs are less likely to cause tolerance and withdrawal due to shorter action and once-daily use (meaning receptors have drug-free periods every day), however, dependence, and withdrawal still commonly occur (Nice, 2004; Pollmann et al., 2015) even after brief or intermittent use (Kales et al., 1991).

### Small dose formulations

Applying these principles in practice requires access to small-dose formulations – tablet cutters and liquid formulations are useful at higher doses. For many drugs, however, even one-quarter the lowest dose of tablet produces large GABA occupancy – e.g. lorazepam (0.125 mg, 5.3% GABA-A occupancy), clonazepam (0.125 mg, 7.8% occupancy), or temazepam (2.5 mg, 5.3% occupancy) (Table 3). For many drugs switching to manufacturer’s liquid formulations may be useful. Another alternative is to use liquids, capsules, or tablets prepared by a compounding pharmacy.

### Off-label options

Off-label methods for small benzodiazepine doses may be appropriate when licensed formulations are unsuitable, consistent with GMC and FDA guidance (General Medical Council, 2026; Office of the Commissioner, n.d.). Many immediate-release, non–enteric-coated tablets can be crushed and dispersed in water without major pharmacokinetic change (Bostwick & Demehri, 2017; Root, Tomlin, Erskine, & Lowey, 2011; Smyth, 2011); diazepam disperses slowly unless crushed (Smyth, 2011). For example, 2 mg diazepam in 20 mL water yields 0.1 mg/mL; thorough mixing before dosing is essential (Colchester Medicines Information, 2018). Without stability data, suspensions should be taken immediately, with any remainder discarded. Some use full-fat milk for an emulsion (greater fat solubility) (Macheras, Koupparis, & Antimisiaris, 1990; White & Bradnam, 2015), also taken immediately. Some patients further dilute existing manufacturers’ liquids with water to reduce the concentration, allowing for smaller incremental dose reductions, or use pipettes to achieve the same effect. Despite practical challenges, patients report these methods as highly useful in withdrawal (Wright, 2020). Others use precision scales to weigh tablet fragments for small reductions.

### Switching to longer-acting benzodiazepines

Evidence does not strongly support or refute switching from shorter- to longer-acting benzodiazepines during tapering (Denis et al., 2005). The rationale is that longer-acting drugs produce more gradual plasma level changes, potentially reducing withdrawal, particularly in those with inter-dose withdrawal from shorter-acting agents, though more frequent dosing of the original drug can also help. Addressing inter-dose withdrawal through one of these two approaches before initiating a taper is wise practice (Horowitz & Taylor, 2024). Notably, drug switching carries risks, due to uncertain bioequivalencies and individual variation in tolerability to different benzodiazepines (Basińska-Szafrańska, 2022).

Diazepam is often chosen for its long half-life (including active metabolites) and availability in small-dose tablets and liquids, but equivalence tables are based on expert opinion, show high inter-individual variability, and should be applied with gradual substitution and a ≥ 2-week stabilization period (Ashton, 2002; Horowitz & Taylor, 2024; Specialist Pharmacy Service, 2022; Wright, 2020). Some patients do not tolerate diazepam well because of sedation and depressed mood (Dhaliwal, Rosani, & Saadabadi, 2025).

In hepatic dysfunction, diazepam may accumulate, and some sensitized patients may require more than once-daily dosing (M. Horowitz & Taylor, 2024). Short half-life z-drugs may produce less receptor adaptation and dependence than drugs with 24-hour coverage, making direct tapering preferable; however, switching may be useful for marked inter-dose withdrawal or failed direct tapering (Horowitz & Taylor, 2024).

### Microtapering

Microtapering involves very small daily dose reductions rather than larger cuts every 1–4 weeks, aiming to minimize homeostatic disruption and reduce withdrawal severity. It requires precise liquid dilutions, small syringes or pipettes, and detailed records, but offers flexibility in pacing, smooths plasma fluctuations, and can be applied to all daily doses or sequentially to individual doses. Rates are calculated by dividing a planned step reduction over the desired days (e.g. 3 mg over 30 days = 0.1 mg/day), with decrements decreasing hyperbolically over time and adjustable to symptoms.

### Use of adjunctive medication

No medications are approved for benzodiazepine withdrawal, and the ASAM panel concluded ‘After carefully considering existing evidence on various pharmacological interventions…no single medication had enough data to support recommending it’ (p. 61) (Brunner et al., 2025), consistent with other analyses (Baandrup et al., 2018; Welsh et al., 2018). Many agents trialed for symptom relief – such as pregabalin, paroxetine, tricyclic antidepressants, and trazodone – can themselves cause dependence and withdrawal, prompting NICE to advise against using dependence-forming medicines (NICE, 2022) and to specifically caution against adding beta-blockers, antidepressants, or antipsychotics where possible (Nice, 2018). Adjusting or reversing the taper is preferable to adding medication, which may cause adverse effects, especially to patients sensitized by withdrawal, and drug interactions (Horowitz & Taylor, 2024). Propranolol and hydroxyzine have been used with inconclusive findings (Nice, 2018). Any adjunct should ideally be short term due to risks of adverse response or dependence – otherwise, the process risks becoming substitution rather than deprescribing (Horowitz & Taylor, 2024).

### Psychological support

Psychological interventions can support benzodiazepine discontinuation, though their effect is modest compared with gradual dose reduction. A meta-analysis of seven studies (454 participants) found adding psychological support to tapering increased cessation rates (OR 1.82) with sustained benefit at follow-up (OR 1.88), whereas gradual tapering alone had a larger effect (OR 6 vs. routine care) (Department of Health, 2017; Nice, 2018). Helpful elements included relaxation training, CBT for insomnia, self-monitoring, goal setting, and anxiety management. CBT does not improve long-term outcomes (Darker et al., 2015) likely because withdrawal is driven by physiological adaptations requiring gradual reduction. Relaxation courses alone improved cessation compared with usual care (Nice, 2018; RxList, 2020). Many patients benefit from understanding withdrawal symptoms as physiological in origin rather than psychological (sometimes called ‘neuro-emotions’) (Framer, 2021; Guy, Davies, & Rizq, 2019). Guidance for therapists advises suspending usual assumptions about distress origins during withdrawal (Guy et al., 2019), as severe affective symptoms may be misdiagnosed as primary mental disorders by clinicians unfamiliar with the process (Framer, 2021). Peer support has been recommended as an effective intervention, typically delivered by individuals with lived experience of benzodiazepine tapering, either in one-on-one or in group settings, in-person or virtually, important for normalization, encouragement, and identification (Lynch et al., 2022; NICE, 2022). Overall, effective tapering aims to minimize distress and functional impairment to patients, mitigating the necessity for additional pharmacologic or therapeutic support.

## Conclusions

Although various regimens are suggested for benzodiazepine tapering, based on pharmacological principles one which reduces dose in a hyperbolic pattern, so as to reduce GABA-A activity in a linear fashion, is most likely to minimize withdrawal severity and increase likelihood of successful cessation. Tapering may require many months or years, in long-term users. In practice, this may involve a test reduction of 5%–10% of the current dose, observing symptoms for 2–4 weeks, and then prescribing a proportionate reduction rate (based on the most recent dose) that is tolerable for the patient, adjusting as necessary. Switching to liquid formulations or other means of making smaller doses may facilitate fine dose adjustments, and many patients will require reducing to very small final doses before complete cessation. Deprescribing guidelines should more accurately reflect hyperbolic benzodiazepine pharmacology. Further research is needed comparing linear and hyperbolic tapering, as well as defining appropriate taper rates based on risk factors. The challenges many patients face in tapering should remain an important consideration and aspect of informed consent when considering prescribing benzodiazepines and z-drugs.

## Long descriptions

### Figure 1. Long description

A three-panel set of line graphs labeled A, B, and C.

Panel A is located at the top-left. The y-axis is Dose of diazepam in m g ranging from 0 to 50. The x-axis is Time in weeks ranging from 0 to 5. A linear decrease is shown starting at 40 m g at week 0 and ending at 0 m g at week 4. Data points are marked at every 10 m g reduction per week.

Panel B is located at the top-right. The y-axis is Dose of diazepam in m g ranging from 0 to 50. The x-axis is Time in weeks ranging from 0 to 60. The data follows a hyperbolic curve. It begins at 40 m g at week 0 and drops rapidly at first, then levels off as it approaches 0 m g near week 52. The rate of reduction slows as the dose gets smaller.

Panel C is located at the bottom-left. The y-axis is Cumulative reduction of diazepam in m g ranging from 0 to 50. The x-axis is Time in weeks ranging from 0 to 60. The curve shows a rapid initial increase in the total amount of drug removed, which then tapers off as it approaches a total reduction of 40 m g by week 52. This curve is an inversion of the hyperbolic pattern seen in Panel B.

Navigate back to Figure 1..

### Table 1. Long description

The table consists of two columns. The first column is titled Diazepam dosage in m g and the second column is titled G A B A dash A occupancy percentage. The data points are as follows.

* 200 mg corresponds to 89.0 percent.

* 100 mg corresponds to 80.2 percent.

* 75 mg corresponds to 75.2 percent.

* 50 mg corresponds to 66.9 percent.

* 37.5 mg corresponds to 60.3 percent.

* 25 mg corresponds to 50.3 percent.

* 12.5 mg corresponds to 33.6 percent.

* 10 mg corresponds to 28.8 percent.

* 5 mg corresponds to 16.8 percent.

* 2 mg corresponds to 7.5 percent.

* 1 mg corresponds to 3.9 percent.

* 0.5 mg corresponds to 2.0 percent.

* 0 mg corresponds to 0 percent.

A footnote indicates that these doses are estimates based on averages with limitations outlined in the text.

Navigate back to Table 1..

### Table 2. Long description

The table consists of four main sections, each containing columns for Step, G A B A-A occupancy percentage, and Diazepam dose mg.

Section 1 (Steps 1 to 21):

* Step 1: 66.9 percent occupancy, 50 mg.

* Step 5: 63 percent occupancy, 42 mg.

* Step 10: 59.3 percent occupancy, 36 mg.

* Step 15: 55.7 percent occupancy, 31 mg.

* Step 21: 50.3 percent occupancy, 25 mg.

Section 2 (Steps 22 to 42):

* Step 22: 49.3 percent occupancy, 24 mg.

* Step 30: 42.2 percent occupancy, 18 mg.

* Step 35: 38.6 percent occupancy, 15.5 mg.

* Step 42: 32.7 percent occupancy, 12 mg.

Section 3 (Steps 43 to 63):

* Step 43: 31.8 percent occupancy, 11.5 mg.

* Step 50: 25.4 percent occupancy, 8.4 mg.

* Step 55: 20.6 percent occupancy, 6.4 mg.

* Step 63: 12.7 percent occupancy, 3.6 mg.

Section 4 (Steps 64 to 81):

* Step 64: 12.1 percent occupancy, 3.4 mg.

* Step 70: 8.2 percent occupancy, 2.2 mg.

* Step 75: 4.6 percent occupancy, 1.2 mg.

* Step 80: 0.8 percent occupancy, 0.2 mg.

* Step 81: 0 percent occupancy, 0 mg.

The data shows a non-linear reduction in dose to achieve a linear reduction in biological effect, with dose reductions becoming smaller as the total dose decreases.

Navigate back to Table 2..

### Table 3. Long description

The table contains 16 rows of drugs with the following column headers: Benzodiazepine or z-drug, Dose equivalent, Elimination half-life in hours, Tablet formulation available, Liquid formulation available, and G A B A occupancy of one-quarter the smallest tablet in percent.

* Alprazolam: 0.25 mg dose; 6 to 12 hour half-life; 0.25 mg and 0.5 mg tablets; 1 mg per m L liquid; 4.8 percent occupancy.

* Chlordiazepoxide: 12.5 mg dose; 5 to 30 hour half-life; 5 mg and 10 mg tablets; liquid N / A; 2.0 percent occupancy.

* Clonazepam: 0.25 mg dose; 18 to 50 hour half-life; 0.5 mg and 2 mg tablets; 0.5 mg per 5 m L and 2 mg per 5 m L liquid; 9.2 percent occupancy.

* Clorazepate: 15 mg dose; 20 to 160 hour half-life; tablets available in U S (3.75, 7.5, 15 mg) and Europe (5, 10, 20, 50 mg); liquid N / A; occupancy is 2.5 percent in U S and 3.3 percent in Europe.

* Diazepam: 5 mg dose; 20 to 100 hour half-life; 2, 5, and 10 mg tablets; 2 mg per 5 m L liquid; 2.0 percent occupancy.

* Eszopiclone: 1 mg dose; 6 hour half-life; 1, 2, and 3 mg tablets; liquid N / A; 3.3 percent occupancy.

* Flurazepam: 7.5 to 15 mg dose; 40 to 250 hour half-life; 15 and 30 mg tablets; liquid N / A; 7.1 percent occupancy.

* Lorazepam: 0.5 mg dose; 10 to 20 hour half-life; 0.5, 1, and 2.5 mg tablets; 1 mg per m L liquid; 4.8 percent occupancy.

* Lormetazepam: 0.5 to 1 mg dose; 10 to 12 hour half-life; 500 micro-grams and 1 mg tablets; liquid N / A; 4.8 percent occupancy.

* Nitrazepam: 5 mg dose; 15 to 38 hour half-life; 5 mg tablets; 2.5 mg per 5 m L liquid; 4.8 percent occupancy.

* Oxazepam: 10 mg dose; 4 to 15 hour half-life; 10 and 15 mg tablets; liquid N / A; 4.8 percent occupancy.

* Temazepam: 10 mg dose; 8 to 22 hour half-life; 10 and 20 mg tablets; 10 mg per 5 m L liquid; 4.8 percent occupancy.

* Triazolam: 0.25 mg dose; 1.5 to 5.5 hour half-life; 0.125 and 0.25 mg tablets; liquid N / A; 3 percent occupancy.

* Zaleplon: 10 mg dose; 1 to 1.5 hour half-life; 5 and 10 mg tablets; liquid N / A; 2.5 percent occupancy.

* Zolpidem: 10 mg dose; 2 hour half-life; 5 and 10 mg tablets; liquid N / A; 4.3 percent occupancy.

* Zopiclone: 7.5 mg dose; 5 to 6 hour half-life; 3.75 and 7.5 mg tablets; liquid N / A; 1.4 percent occupancy.

Navigate back to Table 3..

### Box 2. Long description

At the center of the diagram is a box labeled Benzodiazepine withdrawal syndrome. Arrows point outward from this central box to seven surrounding boxes that categorize specific symptoms.

* Top-Left box: Severe rapid reduction. Symptoms include Catatonia can be fatal, Seizures can be fatal, Delirium tremens, Confusion, Hyperthermia, Mania, Organic brain syndrome, Psychosis e.g. paranoia, Suicidal ideation or suicide, and Violence and aggression.

* Top-Center box: General Somatic. Symptoms include Chest pain, Dry mouth, Gastrointestinal including nausea, diarrhea, vomiting, Headache, Perspiration, Palpitations, Fatigue and weakness, Muscle pain, Sweating, and Elevated blood pressure.

* Top-Right box: Psychological. Symptoms include Agitation and anxiety, Terror and panic attacks, Depersonalisation/derealisation, Depression can be severe, Hypochondriasis, Mood instability, Paranoia, Obsessive compulsive symptoms, Suicidality and self-harm, Irritability, agitation, aggression, and Anxiety.

* Middle-Right box: Sleep Disturbance. Symptoms include Insomnia, Hypersomnia, REM sleep rebound, Nightmares, and Excessive dreaming.

* Lower-Right box: Autonomic. Symptoms include Postural hypotension, Hypertension, and Tachycardia.

* Bottom-Right box: Cognitive. Symptoms include Confusion, Impaired concentration, and Impaired memory.

* Bottom-Center box: Sensory symptoms. Symptoms include Hearing disturbance, Dysesthesia, Neuropathic pain, Tingling, numbness, altered sensation, Visual disturbances, Tinnitus, Sensory hypersensitivity light, sounds, taste, smell, and Paraesthesia.

* Middle-Left box: Neurological/ neuromuscular symptoms. Symptoms include Akathisia, restlessness, Ataxia, Blurred vision, Dilated pupils, Dizziness, Hypnagogic hallucinations, Mild to moderate aphasia, Muscular spasms, cramps, discomfort or fasciculations, Myoclonus, Tremor, Hot and cold spells, Photophobia, Restless legs syndrome, Co-ordination, balance problems, Stiffness, Increased urinary frequency, and Loss of appetite and weight loss.

Navigate back to Box 2..

### Figure 2. Long description

Three line graphs, labeled A, B, and C, share identical axes. The x-axis represents Diazepam dose in milligrams from 0 to 100. The y-axis represents GABA-A occupancy as a percentage from 0 to 100. All three graphs feature a hyperbolic curve that rises steeply from the origin and flattens as it approaches 80 percent occupancy.

* Graph A shows the baseline hyperbolic relationship with data points at approximately 5, 10, 30, 50, 75, and 100 milligrams.

* Graph B illustrates linear dose reductions. Red dashed lines mark equal horizontal intervals on the x-axis at 50, 37.5, 25, and 12.5 milligrams. These correspond to increasingly large vertical drops on the y-axis, showing that equal dose cuts lead to accelerating losses in receptor occupancy.

* Graph C illustrates hyperbolic dose reductions. Red dashed lines mark equal vertical intervals on the y-axis at 80, 60, 40, and 20 percent occupancy. These correspond to progressively smaller horizontal dose reductions on the x-axis, showing that smaller dose cuts at lower levels are required to maintain a linear reduction in receptor effect.

Navigate back to Figure 2..

### Figure 3. Long description

Four panels labeled A through D.

Panel A. Top-left. The X axis is Diazepam concentration in nM from 0 to 400. The Y axis is GABA current percentage from negative 50 to 150. A curve rises sharply from the origin and plateaus near 125 percent at 300 n M.

Panel B. Top-right. The X axis is Triazolam dose in ug/kg from 0 to 300. The Y axis is increase in seizure threshold percentage from negative 200 to 600. The curve rises steeply from 0 and levels off at approximately 500 percent between 200 and 250 ug/kg.

Panel C. Bottom-left. The X axis is Lorazepam dose in mg from 0 to 15. The Y axis is Anxiolysis from 0 to 40. The curve shows a hyperbolic increase, starting at approximately 5 and reaching nearly 30 at a dose of 10 m g.

Panel D. Bottom-right. The X axis is Zolpidem dose in mg from 0 to 25. The Y axis is Total sleep time in minutes from 445 to 470. The curve begins at 447 minutes at zero dose and rises hyperbolically toward 465 minutes at a 20 mg dose.

Navigate back to Figure 3..
